# Health-related quality of life following neoadjuvant chemoradiotherapy versus perioperative chemotherapy and esophagectomy for esophageal cancer: a European multicenter study

**DOI:** 10.1093/dote/doac069

**Published:** 2022-10-14

**Authors:** N Schuring, S R Markar, E R C Hagens, E Jezerskyte, M A G Sprangers, P Lagergren, A Johar, S S Gisbertz, M I van Berge Henegouwen, the LASER study group

**Affiliations:** Department of Surgery, Amsterdam UMC Location University of Amsterdam, Amsterdam, The Netherlands; Cancer Center Amsterdam, Cancer Treatment and Quality of Life, Amsterdam, The Netherlands; Nuffield Department of Surgery, University of Oxford, Oxford, UK; Department of Molecular Medicine & Surgery, Karolinska Institutet, Karolinska University Hospital, Stockholm, Sweden; Department of Surgery, Amsterdam UMC Location University of Amsterdam, Amsterdam, The Netherlands; Cancer Center Amsterdam, Cancer Treatment and Quality of Life, Amsterdam, The Netherlands; Department of Surgery, Amsterdam UMC Location University of Amsterdam, Amsterdam, The Netherlands; Cancer Center Amsterdam, Cancer Treatment and Quality of Life, Amsterdam, The Netherlands; Department of Medical Psychology, Amsterdam UMC Location University of Amsterdam, Amsterdam, The Netherlands; Department of Molecular Medicine & Surgery, Karolinska Institutet, Karolinska University Hospital, Stockholm, Sweden; Department of Surgery and Cancer, Imperial College London, London, UK; Department of Molecular Medicine & Surgery, Karolinska Institutet, Karolinska University Hospital, Stockholm, Sweden; Department of Surgery, Amsterdam UMC Location University of Amsterdam, Amsterdam, The Netherlands; Cancer Center Amsterdam, Cancer Treatment and Quality of Life, Amsterdam, The Netherlands; Department of Surgery, Amsterdam UMC Location University of Amsterdam, Amsterdam, The Netherlands; Cancer Center Amsterdam, Cancer Treatment and Quality of Life, Amsterdam, The Netherlands

**Keywords:** chemotherapy, esophageal cancers, health-related quality of life, neoadjuvant chemoradiation

## Abstract

Curative treatment for locally advanced esophageal cancer consists of (neo)adjuvant treatment followed by esophagectomy. Both neoadjuvant chemoradiotherapy and perioperative chemotherapy improve the 5-year overall survival rate compared with surgery alone. However, it is unknown whether these treatment strategies are associated with differences in long-term health-related quality of life (HRQL). The aim of this study is to compare long-term HRQL in patients after esophagectomy treated with neoadjuvant chemoradiotherapy or perioperative chemotherapy. Disease-free cancer patients having undergone esophagectomy and (neo)adjuvant treatment in one of the participating lasting symptoms after esophageal resection (LASER) study centers between 2010 and 2016, were identified from the LASER study dataset. Included patients completed the European Organisation for Research and Treatment of Cancer Quality of Life Questionnaire C30 (EORTC QLQ-C30), EORTC QLQ-OG25, and LASER questionnaires at least 1 year after the completion of treatment. Long-term HRQL was compared between patients treated with neoadjuvant chemoradiotherapy or perioperative chemotherapy, using univariable and multivariable regression and presented as differences in mean score. Among the 565 included patients, 349 (61.8%) received neoadjuvant chemoradiotherapy, and 216 (38.2%) perioperative chemotherapy. Patients treated with perioperative chemotherapy reported more symptomatology for diarrhea (*difference in means 5.93*), reflux (*difference in means 7.40*), and odynophagia (*difference in means 4.66*). The differences did not exceed the 10 points to be of clinical relevance. No significant differences for the LASER key symptoms were observed. The observed differences in long-term HRQL are in favor of patients treated with neoadjuvant chemoradiotherapy compared with patients treated with perioperative chemotherapy; however, the differences were small. Patients need to be informed about long-term HRQL when considering allocation of (neo)adjuvant treatment.

## INTRODUCTION

Esophageal cancer is increasing in incidence. In 2020, over 600,000 patients were diagnosed with esophageal cancer worldwide.^1^ In the past three decades, the survival rates of esophageal cancer have improved from around 10% to almost 50%.^2–6^ This improvement is partly explained by the contribution of multimodal treatment. Two commonly used (neo)adjuvant strategies are neoadjuvant chemoradiotherapy and perioperative chemotherapy. The CROSS trial, comparing the 5-year survival rate between patients treated with neoadjuvant chemoradiotherapy plus surgery with patients treated with surgery alone, found survival percentages of 47 and 33%, respectively.^5^ Similar improvements in 5-years survival were found in the MAGIC trial, investigating the survival of patients with resectable adenocarcinoma of the stomach, gastroesophageal junction, or lower esophagus who were either treated with a combination of surgery and perioperative chemotherapy or surgery alone. The 5-year survival rate was 36.3 and 23.0%, respectively.^3^^,^^5^ Several studies compared both neoadjuvant chemoradiotherapy and perioperative chemotherapy, with regard to, among others, survival, postoperative complications, toxicity, and pathological response. So far no oncologic superiority of either strategies has been demonstrated; however, neoadjuvant chemoradiotherapy has been found to be associated with a lower incidence of severe adverse events.^7–12^

With the improvements in long-term survival for esophagectomy patients, the effect of treatment on short and long-term health-related quality of life (HRQL) has become more compelling. HRQL following esophagectomy was found to be poorer than in the general population.^13^ The decline in HRQL is temporary as it restores to baseline in disease-free patients within 2 years after surgery.^14–17^ A recent study comparing HRQL in patients treated after upfront surgery with patients receiving neoadjuvant chemoradiation followed by surgery showed that HRQL declined during neoadjuvant chemoradiotherapy, but did not significantly differ between the groups postoperatively.^18^ The NeoRes trial is the only randomized trial (*n* = 165) comparing the short- and long-term HRQL in patients randomized to perioperative chemotherapy or neoadjuvant chemoradiotherapy from 10 centers in Sweden and Norway. A large deterioration in HRQL was observed at 1 year, and recovery was seen at 3 and 5 years in both arms. The only clinically relevant difference was that patients treated with neoadjuvant chemotherapy reported more odynophagia shortly after completion of neoadjuvant treatment, and more problems with coughing at 3 years.^19^

The primary aim of the current European multicenter study is to examine whether the long-term HRQL differs between esophageal cancer patients treated with neoadjuvant chemoradiotherapy versus perioperative chemotherapy followed by an esophagectomy who participated in the LASER study.^20–22^ We hypothesized that patients treated with neoadjuvant chemoradiotherapy would have a less impaired HRQL, as neoadjuvant chemoradiotherapy is associated with a lower risk of adverse events.^12^ The secondary aim is to compare the HRQL of each (neo)adjuvant therapy group with that of the general population.

## METHODS

### The lasting symptoms after esophageal resection study database

This cohort study was performed with the data from the multicenter LASER (lasting symptoms after esophageal resection) study.^20^ Informed consent was obtained from all participating patients to use their data in retrospective studies. Ethical approval was gained by each participating center during the LASER study to use the LASER study dataset for side studies. The STROBE guidelines were followed for the structure of this article.^23^

The LASER study is a multi-center cross-sectional study, including patients from 20 European centers from seven countries, which aimed to identify the most prevalent symptoms and those with greatest impact on HRQL among disease-free patients following an esophagectomy for esophageal or junctional (Siewert I and II) cancer.^20^ Patients completed three HRQL questionnaires once (the European Organisation for Research and Treatment of Cancer Quality of Life Questionnaire C30 [EORTC QLQ-C30], EORTC QLQ-OG25, and the LASER symptom questionnaire).^20^

### Patient, tumor, and treatment characteristics

Collected characteristics included age, sex, (neo) adjuvant therapy (neoadjuvant chemoradiotherapy or perioperative chemotherapy), tumor location, surgical techniques (Ivor Lewis, McKeown, transhiatal, left thoracoabdominal), surgical access (minimally invasive esophagectomy (MIE), hybrid, open), location of anastomosis (cervical or intrathoracic), and pathological stage (0, I, II, III–IV).

#### Questionnaires

HRQL was measured with the validated EORTC QLQ-C30 and EORTC QLQ-OG25 questionnaires. Combined, they assess 31 domains, and outcome scores were linearly transformed in scores ranging from 0 to 100. Higher scores correspond to better HRQL in the functioning domains and for global health, whereas higher scores represent more symptomatology or a larger negative effect for symptom domains and financial difficulties.^21^^,^^22^

In addition, the LASER questionnaire, developed by Markar *et al*., assessing the most prevalent and impactful symptoms in patients surviving >1 year after esophagectomy for cancer, was used.^20^ The questionnaire assesses, among others, 23 symptoms. Three symptoms, as previously described in the study by Markar *et al*., were identified to be associated with a poor HRQL; pain from scars on chest, low mood, and reduced energy or activity tolerance. These three symptoms will be investigated in this study and will be referred to as LASER key symptoms. The LASER key symptoms were graded with a composite score from 0 to 5, with 5 indicating more symptomatology (Appendix A).^20^ For more detailed information, we refer to the EORTC scoring manual, the EORTC website, and the original LASER study article.^20–22^

**Appendix A:** Grading system for each LASER symptom according to the patient-reported impact upon quality of life (QOL) and frequency of the symptom.^20^

**Symptom level**	**QOL impact and frequency**
0	No symptom present
1	QOL impact = None
2	QOL impact = Some & Frequency = rarely/weekly
3	QOL impact = Some & Frequency = daily/multiple
4	QOL impact = Substantial & Frequency = rarely/weekly
5	QOL impact = Substantial & Frequency = daily/multiple

#### Population reference values

To compare the patients’ responses to the EORTC QLQ-C30 and the EORTC QLQ-OG25 with those of the general population, the reference values for the EORTC QLQ-C30 provided in the EORTC reference manual and the reference values for the EORTC QLQ-OG25 published by van der Schaaf *et al*. will be used.^24^^,^^25^ The reference values of the EORTC reference manual are based on a sample of 7.802 healthy persons (52% male and 48% female) from the general population from Germany, Norway, Austria, Denmark, and the USA, and those by van der Schaaf *et al*. on a random sample of 4910 persons (age ranging from 40 to 79 years; 69.97% males and 30.1.% females) from the Swedish population.

### Statistical analysis

Patient demographics, tumor, treatment, and pathological data and HRQL scores were presented using descriptive statistics. Continuous variables are presented as means with standard deviation (SD) and categorical variables as frequencies and percentages.

Univariable and multivariable linear regression analyses were performed to test the differences in HRQL domain scores (EORTC QLQ-C30 and EORTC QLQ-OG25) between patients treated with neoadjuvant chemoradiotherapy and patients treated with perioperative chemotherapy. The possible confounding effect of ‘age’ was adjusted in the model, as age was considered as the only variable associated with both exposures and outcomes. To reduce multiple testing, multivariate analysis was only performed if a *P-*value of <0.10 was reached in univariable analysis. Mean scores with 95% confidence intervals (95% CI) were calculated and compared between the two separate groups. A *P-*value of <0.01 was considered statistically significant. A difference between means of 10 points or more was considered clinically relevant.^21^^,^^22^^,^^26^^,^^27^

For the three LASER key symptoms, odds ratios (OR) with a 95% CI were calculated, using logistic regression models, between the separate groups. Again, the model was adjusted for ‘age’ and to reduce multiple testing, multivariate analysis was only performed if a *P-*value of <0.10 was reached in univariable analysis. A *P-*value of <0.01 was considered statistically significant.

For the second aim, we compared the mean outcomes of each HRQL domain of the study population, specified for patients treated with neoadjuvant chemoradiotherapy and patients treated with perioperative chemotherapy, with those of the general population.^24^^,^^25^ Again, a score difference of at least 10 points were considered to be clinically meaningful.

All data were handled anonymously. Statistical analyses were conducted by an experienced biostatistician (A.J.) and performed using SAS 9.4 software.

## RESULTS

### Patient, tumor, and treatment characteristics

**Fig. 1:**
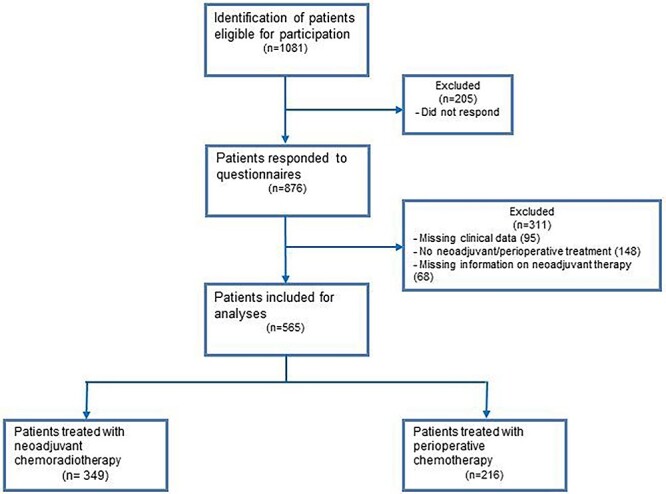
Study flow chart LASER cohort.

In total, 876 of 1081 invited patients (response rate 81%) participated in the LASER study. Hundred forty-eight patients did not receive (neo)adjuvant therapy, of 95 patients, clinical data were missing, and of 68 patients, information on what kind of (neo)adjuvant therapy they received was missing. These patients were therefore excluded, leaving 565 patients for analysis (Fig. 1).

The majority of the included patients were male (78.8%), the mean age was 63.7 years (SD 8.6), and the average number of years since completion of therapy was 4.3 years (SD 1.7). Three hundred and forty-nine patients (62%) received neoadjuvant chemoradiotherapy and 216 (38%) received perioperative chemotherapy.

This was a cross-sectional study design and therefore there was a one-time assessment of the HRQL. The patients eligible for participation in the study were at least 1 year after esophagectomy. The mean time since surgery was 4.3 years (SD 1.7) for the total study population, 4.2 years (SD 1.6) for patients after neoadjuvant chemoradiotherapy, and 4.6 years (SD 1.7) after perioperative chemotherapy. Patient demographics, tumor, specific timing of assessment of HRQL questionnaires and treatment characteristics are presented in Table 1, Supplementary Tables S1–2.

**Table 1:** Patient, tumor, and treatment characteristics

		**Total**		**Neoadjuvant chemoradiotherapy**		**Perioperative chemotherapy**	
		***n* = 565**		***n* = 349**		***n* = 216**	
Age (mean [SD])		63.7	8.6	63.9	8.4	63.5	8.4
Years since surgery (mean, [SD])		4.3	1.7	4.2	1.6	4.6	1.7
Sex (%)	**Male**	445	21.2%	278	79.6%	167	77.3%
	**Female**	120	78.8%	71	20.3%	49	22.7%
Surgical technique (%)	**Ivor Lewis**	310	54.9%	172	49.3%	138	63.9%
	**Left thoracicoabdominal**	41	7.3%	0	-	41	19%
	**McKeown**	142	25.1%	114	32.7%	28	13%
	**Transhiatal**	72	12.7%	63	18.1%	9	4.2%
Surgical access (%)	**Hybrid**	102	18.1%	71	20.3%	31	14.4%
	**MIE**	153	27.1%	112	32.1%	41	19%
	**Open**	310	54.0%	166	47.6%	144	66.7%
Location anastomosis (%)	**Cervical**	215	38.0%	181	51.9%	34	15.7%
	**Intrathoracic**	350	62.0%	168	48.1%	182	84.3%
Country (%)	**Netherlands**	190	33.6%	168	48.1%	22	10.2%
	**United Kingdom**	151	26.7%	11	3.2%	140	64.8%
	**Sweden**	55	9.7%	41	11.7%	14	6.5%
	**France**	22	3.9%	6	1.7%	16	7.4%
	**Italy**	71	12.6%	61	17.5%	10	4.6%
	**Ireland**	44	7.8%	34	9.7%	10	4.6%
	**Spain**	31	5.5%	27	7.7%	4	1.9%
	**Missing**	1	0.2%	1	0.3%	-	-
Pathological stage (%)	**0**	127	22.4%	109	31.2%	18	8.3%
	**I**	129	22.8%	92	26.4%	37	17.1%
	**II**	153	27.1%	75	21.5%	78	36.1%
	**III-IV**	129	22.8%	54	15.5%	75	34.7%
	**Missing**	27	4.8%	19	5.4%	8	3.7%

SD = standard deviation, percentages may not add up to 100 due to rounding.

### Perioperative chemotherapy versus neoadjuvant chemoradiotherapy

After univariable linear regression analysis for all EORTC QLQ-C30 domains, a *P*-value of <0.10 was found for emotional functioning, social functioning, fatigue, pain, insomnia, and diarrhea. After multivariable regression, only the difference in means for diarrhea (difference in means 5.93, *P =* 0.009) was statistically significant in favor of the neoadjuvant chemoradiotherapy group. This difference was not clinically relevant, as it did not reach the threshold of 10 points (Table 2).

**Table 2:** Univariable and multivariable analyses EORTC QLQ-C30 and EORTC QLQ-OG25 questionnaire

			**Univariable analysis**				**Multivariable analysis^**			
	**Neoadjuvant chemoradiotherapy mean (95% CI)**	**Perioperative chemotherapy mean (95% CI)**	**Difference in means**	**95% CI**		***P*-value**	**Difference in means**	**95% CI**		***P*-value**
	***n* = 349**	***n* = 216**		**Lower**	**Upper**			**Lower**	**Upper**	
**EORTC QLQ-C30**										
Global Health	74.0 (71.8–76.2)	71.7 (68.9–74.5)	−2.3	−5.9	1.2	0.20				
**Functioning**										
Physical functioning	82.9 (81.0–84.9)	82.7 (80.2–85.2)	−0.2	−3.4	2.9	0.89				
Role functioning	80.7 (78.0–83.4)	80.0 (76.5–83.5)	−0.7	−5.1	3.8	0.77				
Emotional functioning	83.7 (81.4–86.0)	79.3 (76.4–82.3)	−4.4	−8.1	−0.7	0.02^*^	−4.0	−8.2	0.3	0.06
Cognitive functioning	84.7 (82.5–86.9)	82.0 (79.2–84.8)	−2.7	−6.3	0.9	0.14				
Social functioning	83.8 (81.2–86.4)	77.6 (74.3–80.9)	−6.2	−10.4	−2.0	**<0.005** ^*^	−4.6	−9.1	0.0	0.05
**Symptom scores**										
Fatigue	27.2 (24.6–29.7)	30.9 (27.6–34.2)	3.8	−0.4	7.9	0.08^*^	2.8	−2.0	7.5	0.25
Nausea and vomiting	12.4 (10.4–14.4)	12.4 (9.9–15.0)	0.1	−3.2	3.3	0.98				
Pain	13.1 (10.8–15.3)	16.2 (13.3–19.1)	3.1	−0.5	6.8	0.09^*^	3.9	−0.1	7.9	0.06
Dyspnoea	21.5 (18.6–24.4)	21.8 (18.0–25.5)	0.3	−4.3	5.0	0.91				
Insomnia	20.4 (17.4–23.3)	26.9(23.1–30.6)	6.5	1.8	11.3	**<0.005** ^*^	5.7	0.4	10.9	0.04
Appetite loss	15.5 (12.8–18.2)	17.7 (14.2–21.2)	2.2	−2.3	6.6	0.33				
Constipation	10.0 (7.9–12.2)	13.0 (10.2–15.7)	2.9	−0.6	6.4	0.10				
Diarrhea	13.7 (11.3–16.2)	20.3 (17.2–23.4)	6.6	2.6	10.5	**<0.005** ^*^	5.9	1.5	10.4	**<0.01**
**Financial**										
Financial difficulties	12.9 (10.1–15.7)	14.1 (10.5–17.6)	1.2	−3.4	5.7	0.61				
**EORTC QLQ-OG25**										
**Mutliple item**										
Dysphagia	9.3 (7.5–11.0)	10.5 (8.3–12.8)	1.3	−1.6	4.1	0.38				
Eating restrictions	21.0 (18.7–23.3)	23.3 (20.3–26.3)	2.3	−1.5	6.1	0.23				
Reflux	24.1 (21.3–26.9)	30.0 (26.4–33.7)	6.0	1.4	10.5	0.01^*^	7.4	2.0	12.8	**<0.01**
Odynophagia	8.5 (6.8–10.4)	12.3 (10.1–14.4)	3.8	1.1	6.6	**0.01** ^*^	4.7	1.5	7.8	**<0.01**
Pain and discomfort	14.5 (12.2–16.7)	19.6 (16.7–22.4)	5.1	1.4	8.7	**0.01** ^*^	4.3	0.2	8.5	0.04
Anxiety	26.7 (23.7–29.6)	30.9 (27.2–34.7)	4.3	−0.5	9.0	0.08^*^	4.2	−1.1	9.4	0.12
**Single item**										
Eating with others	13.5 (10.9–16.1)	12.0 (8.7–15.3)	−1.5	−5.7	2.7	0.48				
Dry mouth	20.1 (17.1–23.1)	24.0 (20.2–27.9)	4.0	−0.9	8.8	0.11				
Trouble with taste	13.1 (10.4–15.7)	14.3 (10.9–17.6)	1.2	−3.1	5.5	0.58				
Body image	13.4 (10.7–16.0)	14.5 (11.0–17.9)	1.1	−3.2	5.5	0.61				
Trouble swallowing saliva	6.5 (4.8–8.3)	5.6 (3.4–7.9)	−0.9	−3.8	2.0	0.54				
Choked when swallowing	10.8 (8.7–12.8)	9.9 (7.2–12.5)	−0.9	−4.3	2.5	0.61				
Trouble with coughing	29.2 (26.3–32.2)	30.6 (26.7–34.4)	1.3	−3.5	6.2	0.59				
Trouble talking	10.3 (8.2–12.5)	8.3 (5.5–11.0)	−2.1	−5.5	1.4	0.24				
Weight loss	18.5 (15.5–21.5)	18.4 (14.5–22.3)	−0.1	−5.0	4.9	0.98				
Hair loss	25.6 (24.1–27.1)	26.7 (24.8–28.6)	1.1	−1.3	3.5	0.35				

Difference in means (MD) with 95% confidence interval (CI) are shown for univariable and multivariable analysis. ^= corrected for confounders. *= Health related quality of life (HRQL) domains with *p*-value < 0.1 in univariable analysis were entered in multivariable analysis. In bold values that were statistically significant. (*p*-value < 0.01).

**Fig. 2:**
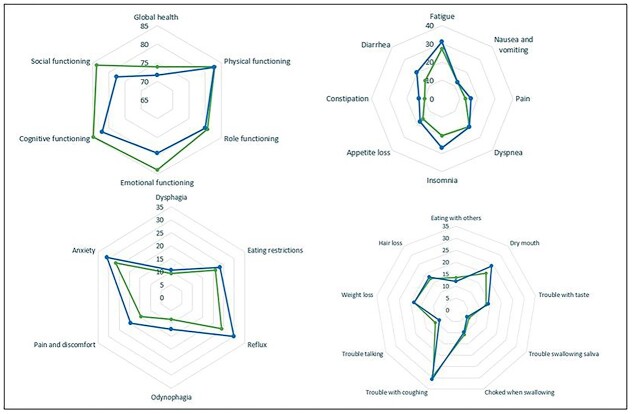
Spider plot showing domain outcome scores for patients treated with ‘neoadjuvant chemoradiotherapy’ (green line) and patients treated with ‘perioperative chemotherapy’ (blue line). A: EORTC QLQ-C30 Global health and functioning domains, B: EORTC QLQ-C30 symptom and financial domains, C: EORTC QLQ-OG25 Multiple-item scale, D: EORTC QLQ-OG25 Single-item scales.

For the EORTC QLQ-OG25 domains, reflux, odynophagia, pain and discomfort, and anxiety, a *P*-value of <0.10 was found after univariable linear regression analysis. In multivariable analysis, significantly more symptomatology for reflux (difference in means 7.40, *P =* 0.007) and odynophagia (difference in means 4.66, *P =* 0.004) was found in the perioperative chemotherapy group. However, none of these results were clinically relevant (Table 2 and Fig. 2).

The following ORs were found for the LASER key symptoms pain from scars on chest (OR 1.05, *P =* 0.887), low mood (OR 1.4, *P =* 0.219), and reduced energy or activity tolerance (OR 0.71, *P =* 0.078) after univariable analysis. Multivariate analysis was only performed for reduced energy or activity tolerance (OR 0.77, *P =* 0.23), as this was the only LASER key symptom that reached the threshold for multivariable analysis. None of the ORs found after univariable or multivariable analysis was statistically significant (Table 3).^20^

**Table 3:** Univariable and multivariable analyses of the LASER key symptoms comparing patient receiving perioperative chemotherapy and neoadjuvant chemoradiotherapy

	**Univariable analysis**		**Multivariable analysis**	
	**OR (95% CI)**	***P-*value**	**OR (95% CI)**	***P*-value**
**Pain from scars on your chest**	1.05 (0.51–2.16)	0.89	1.3	0.53
**Low mood**	1.43 (0.81–2.54)	0.22	1.64	0.13
**Reduced energy/activity tolerance**	0.71 (0.49–1.04)	0.08^*^	0.77 (0.50–1.18)	0.23

Odds ratio comparing patients treated with ‘perioperative chemotherapy’ versus ‘neoadjuvant chemoradiotherapy’ with 95% confidence interval (CI) are shown for univariable analysis and multivariable analysis

^*^LASER key symptoms with *P*-value <0.1 in univariable analysis were entered in multivariable analysis. In bold values that were statistically significant (*P*-value <0.01)

### Esophageal cancer patients versus general population

Patients reported more symptomology compared with the general population on most domains. This was more pronounced in patients treated with perioperative chemotherapy. Results are displayed in Supplementary Table S2A.^24^^,^^25^

## DISCUSSION

This study investigated the differences in long-term HRQL and LASER key symptoms between patients following neoadjuvant chemoradiotherapy and perioperative chemotherapy and esophagectomy for esophageal or gastroesophageal junction cancer. Our results show that all the statistically significant differences found for the long-term HRQL domains (diarrhea, reflux, and odynophagia) between patients treated with neoadjuvant chemoradiotherapy and perioperative chemotherapy after a mean time of 4.3 years after treatment, are in favor of patients treated with neoadjuvant chemoradiotherapy. However, none of these differences reached the threshold for clinical relevance. In addition, compared with the reference values of the general population, patients treated with perioperative chemotherapy scored poorer on more domains than patients treated with neoadjuvant chemoradiotherapy. This is in line with our hypothesis that neoadjuvant chemoradiotherapy might impair the long-term HRQL less than perioperative chemotherapy.

Several studies have reported that the post-operative reduction in HRQL is restored after 1–2 years in disease-free patients.^14^^,^^28^ In the study by Jezerskyte *et al*., investigating HRQL between patients with and without complications after multimodal treatment for esophageal cancer at different time points, a significant decline in short-term HRQL was found, which restored to the baseline level during the 12-month follow-up period.^17^ In the study by Blazeby *et al*., similar results were found; a deterioration in HRQL was found in esophageal cancer patients treated with multimodality therapy, which recovered after 6–9 months.^15^ Similar post-operative HRQL scores for patients who had undergone surgery alone (*n* = 21), and patients who received multimodal therapy (neoadjuvant chemoradiotherapy [*n* = 34] or perioperative chemotherapy [*n* = 48] followed by an esophagectomy) were observed.^15^ Surprisingly, patients who were treated with surgery alone had a delayed recovery for symptom domains nausea and emesis, and dysphagia compared with patients treated with neoadjuvant therapy followed by surgery. The total sample size (*n* = 103) of this study was quite small and therefore may have lacked power to detect differences between patients of the two multimodal therapy groups. In a small cohort study (*n* = 87) conducted by Djärv *et al*., no improvements in HRQL between 6 months and 3 years after esophagectomy with curative intent were observed. Worse outcomes for the domains role functioning, social functioning, fatigue, diarrhea, appetite loss, and vomiting compared with the reference population were found at 3 years after esophagectomy. In this study, details about (neo)adjuvant therapy were not provided and the influence of (neo)adjuvant treatment was not investigated.^13^ Anderegg *et al*. compared patients treated with neoadjuvant chemoradiotherapy or perioperative chemotherapy, and investigated among other oncological outcomes and the toxicity of the (neo)adjuvant treatment schemes. In this study, treatment with neoadjuvant chemoradiotherapy or perioperative chemotherapy lead to equal oncological outcomes (radical resection rates, lymphadenectomy, patterns of recurrent disease, and [disease-free] survival). The risk of serious adverse events and the necessity to interrupt treatment were significantly higher for the patients treated with perioperative chemotherapy.^12^ In the previously described NeoRes trial, the majority of the found differences in long-term HRQL between perioperative chemotherapy and neoadjuvant chemoradiotherapy were not clinically relevant and statistically significant. Interestingly, patients in both arms reported a deterioration of HRQL after surgery, which recovered over time, but more symptomatology remained in the neoadjuvant chemoradiotherapy group. This discrepancy with our results might be explained by the fact that the neoadjuvant chemoradiotherapy regimen in the NeoRes trial was before the broad implementation of the CROSS scheme, and consisted of more intense chemotherapy (cisplatin and 5-fluorouracil +40 Gy in 2-Gy fractions).^19^

Limitations and strengths of this study should be emphasized. First, because of the primary limitation of a cross-sectional study design, in this case solely a one-time assessment of the HRQL questionnaires, it is not possible to derive a causal relationship between the exposure, type of (neo)adjuvant therapy, and the HRQL. In addition, due to the one-time assessment, we do not know the pre-treatment HRQL and LASER key symptom scores, nor the baseline patients’ characteristics, therefore we cannot report changes in time or observe specific trends. Moreover, only disease-free patients were included in this study, therefore the results are not representative for all patients following an esophagectomy for esophageal or junctional cancer, as nearly half of all patients will develop a recurrence, predominantly in the first year after surgery.^29^

Second, several significant differences in means were observed; however, they were deemed to be not clinically relevant, given earlier guidelines that defined a mean difference of 10 points or more to indicate a clinically relevant difference.^26^^,^^27^ However, there is increasing evidence that the minimal difference indicative of clinical relevance differs per scale, direction, clinical setting and disease, and that we should not expect one single minimally important difference for the different scales/domains of the different questionnaires.^26^^,^^30–34^ Musoro *et al*. investigated thresholds for minimally clinical significant change for patients with a malignant melanoma, advanced breast cancer, head and neck cancer, and ovarian cancer and unfortunately not for esophageal cancer patients. They found the minimally important change of scores over time to vary between 4 and 10 point for most scales.^32–35^ As Osoba *et al*. already noted in 1998, a difference of 5–10 points would indicate ‘a little’ change. If we would have taken for instance, a 5-point difference in means as cut-off value for a clinically relevant difference, the statistically significant differences in means found for domains diarrhea and reflux would have been clinically relevant, and in favor for patients treated with neoadjuvant chemoradiotherapy.^26^

Another point to address is the difference in surgical technique and location of the anastomosis between the neoadjuvant chemoradiotherapy and perioperative chemotherapy groups, with 48 and 84.3%, respectively, having an intrathoracic anastomosis and the remaining patients receiving a cervical anastomosis. In cervical compared with intrathoracic anastomosis, the incidence of recurrent laryngeal nerve palsy has shown to be higher, which might influence the domains trouble talking, trouble swallowing saliva, and choked when swallowing.^36^ We observe a trend for more symptomatology for these domains in patients treated with neoadjuvant chemoradiotherapy; however, since information on the incidence of recurrent laryngeal nerve palsy is not available in the LASER dataset, we do not know if these observed differences are attributable to more patients having laryngeal nerve problems in this group. Moreover, the two groups also differed with regard to pathological state, with the perioperative chemotherapy group and neoadjuvant chemoradiotherapy presenting with 70.8 versus 37.0% pathological stage of ≥ II. Unfortunately, we do not have the information on clinical stage or tumor regressions scores of the patients within the two groups, nor was any data collected about the differences in rehabilitation received in the postoperative course. This could have influenced the outcomes, as rehabilitation might have a positive effect on HRQL. These baseline differences may also have affected HRQL in addition to the different (neo)adjuvant treatments.

The main strengths of this study are the large sample size of patients from different cultures, countries, and centers. This diversity in included patients makes the results applicable to esophageal cancer patients who had an esophagectomy in other countries in Europe. Another strength is the high response rate, 81%, of the invited patients who filled out the questionnaires.

*In conclusion,* the observed differences in several long-term HRQL domains between patients treated with perioperative chemotherapy or neoadjuvant chemoradiotherapy followed by an esophagectomy for esophageal or junctional cancer at the mean time of 4.3 years after surgery are in favor of patients treated with neoadjuvant chemoradiotherapy. These differences are small and none reached the threshold for clinical relevance. Patients need to be informed about the long-term HRQL when considering allocation of (neo)adjuvant treatment.

### List of collaborators

**Collaborators**

Sheraz R Markar,^1, 2^ Giovanni Zaninotto,^1^ Carlo Castoro,^3, 4^ Asif Johar,^2^ Pernilla Lagergren,^1, 2^ Jessie A Elliott,^5^ Suzanne S Gisbertz,^6^ Christophe Mariette,^7^ Rita Alfieri,^3^ Jeremy Huddy,^1^ Viknesh Sounderajah,^1^ Eleonora Pinto,^3^ Marco Scarpa,^3^ Fredrik Klevebro,^2^ Berit Sunde,^2^ Conor F Murphy,^5^ Christine Greene,^5^ Narayanasamy Ravi,^5^ Guillaume Piessen,^7^ Hylke Brenkman,^8^ Jelle P Ruurda,^8^ Richard Van Hillegersberg,^8^ Sjoerd Lagarde,^9^ Bas Wijnhoven,^9^ Manuel Pera,^10^ José Roig,^10^ Sandra Castro,^10^ Robert Matthijsen,^11^ John Findlay,^12^ Stefan Antonowicz,^12^ Nick Maynard,^12^ Orla McCormack,^13^ Arun Ariyarathenam,^14^ Grant Sanders,^14^ Edward Cheong,^15^ Shameen Jaunoo,^16^ William Allum,^13^ Jan Van Lanschot,^9^ Magnus Nilsson,^17^ John V Reynolds,^5^ Mark I van Berge Henegouwen,^6^ George B Hanna^1^.

**Affiliations.**

^1^Department Surgery and Cancer, Imperial College London, UK.

^2^Department of Molecular Medicine and Surgery, Karolinska Institutet, Stockholm, Sweden.

^3^Unit of Surgical Oncology of the Esophagus and Digestive Tract, Veneto Institute of Oncology, Padova, Italy.

^4^Division of Upper Gastrointestinal Surgery, Department of Surgery, Humanitas Research Hospital IRCCS, Humanitas University, Rozzano, Milan, Italy.

^5^Department of Surgery, Trinity Centre for Health Sciences, St. James’s Hospital and Trinity College Dublin, Dublin, Ireland.

^6^Department of Surgery, Amsterdam UMC, location AMC, University of Amsterdam, Cancer Center Amsterdam, Amsterdam, the Netherlands.

^7^University Lille, Department of Digestive and Oncological Surgery, Claude Huriez University Hospital, Lille, France.

^8^Department of Surgery, University Medical Center Utrecht, Utrecht, the Netherlands.

^9^Department of Surgery, Erasmus MC, University Medical Center, Rotterdam, the Netherlands.

^10^Department of Surgery, Hospital Vall d’Hebron, Barcelona, Spain.

^11^Department of Gastrointestinal Surgery, ETZ Tilburg, the Netherlands.

^12^Department of Upper GI Surgery, Oxford University Hospitals NHS Foundation Trust, Oxford, UK.

^13^Academic Department of Surgery, Royal Marsden Hospital, London, UK.

^14^Department of Oesophago-Gastric Surgery, Plymouth Hospitals NHS Trust, Plymouth, UK.

^15^Department of Upper Gastrointestinal Surgery, Norfolk & Norwich Hospitals NHS Trust, Norwich, UK.

^16^Department of Upper Gastrointestinal Surgery, Gloucestershire Hospitals NHS Foundation Trust, Gloucestershire, UK.

^17^Department of Clinical Science, Intervention and Technology, Karolinska Institutet and Department of Upper Abdominal Diseases, Karolinska University Hospital, Stockholm, Sweden.

## Funding

M.I. van Berge Henegouwen has a consultant role with Mylan, Johnson and Johnson, Alesi Surgical, B. Braun and Medtronic. Research funding from Stryker.

S.R. Markar received the European Society for Medical Oncology Clinical Research Fellowship for the support of this study. S.R. Markar is supported by an NIHR Academic Clinical Lectureship and acknowledges support from the National Institute for Health Research (NIHR) Imperial Biomedical Research Centre (BRC). P. Lagergren is supported by the National Institute for Health Research (NIHR) Imperial Biomedical Research Centre (BRC) for her position at Imperial College London, London, UK. This LASER study was supported by the NIHR London IVD Co-operative and the Morgagni Charity. The views expressed are those of the authors and not necessarily those of the NHS, the NIHR, or the Department of Health.

## Conflicts of Interest

The authors report no conflicts of interest.

## Supplementary Material

Supplementary_material_doac069
