# Supplementary material for: Health-related quality of life following neoadjuvant chemoradiotherapy versus perioperative chemotherapy and esophagectomy for esophageal cancer: a European multicenter study
Source: Dis Esophagus. 2022 Oct 14;36(4):doac069. doi: 10.1093/dote/doac069 (PMC10061418; doi:10.1093/dote/doac069)
Supplement: Supplementary_material_doac069 [file supplementary_material_doac069.docx]

# Supplementary material

## Table S 1 Details on (neo)adjuvant therapy regimens.

### ***Table S 1a.*** *Details on perioperative chemotherapy regimens.*

| Neoadjuvant chemotherapy | Scheme | Details | Frequencies | Cumulative frequencies |
| --- | --- | --- | --- | --- |
|  | Cisplatin + 5 FU (CF) | 3 cycles  2 cycles  5 cycles  Unknown | 4  3  1  19 | 27 |
|  | Cisplatin + Cetuximab | 6 cycles | 1 | 1 |
|  | Cisplatin + Capecitabine (CX) | Unknown | 7 | 7 |
|  | Cisplatin | 2 cycles  unknown | 1  1 | 2 |
|  | Cisplatin + Docetaxel + 5FU (DCF) | Unknown | 2 | 2 |
|  | Cisplatin + Docetaxel |  | 1 | 1 |
|  | Carboplatin + Paclitaxel | Unknown | 2 | 2 |
|  | Epirubicin, Oxaplatin, Capecitabine + Oxaplatin, 5FU (EOX + FOLFOX) | Unkown | 2 | 2 |
|  | Epirubicin + Cisplatin + Capecitabine (ECC of ECX) | Unknown  4 cycles  3 cycles | 22  1  2 | 25 |
|  | Epirubicin + Oxaplatin + 5FU (EOF) | Unkown | 1 | 1 |
|  | Epirubicin + Oxaplatin + Capecitabine (EOX) | Unknown  1 cycle  2 cycles  3 cycles  6 (3 neoadjuvant, 3 adjuvant) | 17  1  1  4  1 | 24 |
|  | Oxaplatin + 5FU (FOLFOX) | Unknown  4 neo, 2 adjuvant  4 cycles  6 cycles  4 neo, 4 adjuvant | 5  1  1  2  2 | 11 |
|  | Oxaplatin + 5FU+ Herceptin **(**FOLFOX+ Herceptin) | 4 cycles | 1 | 1 |
|  | Epirubicin + Cisplatin + 5FU (MAGIC) | 3 neo, 3 adjuvant | 64 | 64 |
|  | Epirubicin + cisplatin + 5FU (ECF) | Unknown | 3 | 3 |
|  | Trastuzumab and 5FU-DDP (TCF) | TCF 1 cycle, Trastuzumab and 5FU-DDP (5 cycles) | 1 | 1 |
|  | Unknown |  | 42 | 42 |
| Total |  |  |  | **216** |

5 FU= 5-Fluorouracil, CF=cisplatin/5-fluorouracil, CX=cisplatin/capecitabine, TCF or DCF=docetaxel,/cisplatin/5-fluorouracil, EOX=epirubicin/oxaplatin/capecitabine, FOLFOX=folinic acid/fluoracil/oxiplatin, ECC= epirubicin/cisplatin/capecitabine, ECX=epirubicin/cisplatin/capecitabine, EOF=epirubicin/oxaplatin/fluorouracil, ECF=Epirubicin/cisplatin/fluorouracil,

### ***Table S 1b.*** *Details neoadjuvant chemoradiotherapy regime.*

| Neoadjuvant chemoradiotherapy | Scheme | Details | Frequencies | Cumulative frequencies |
| --- | --- | --- | --- | --- |
|  | 5FU + Cisplatin + RT | Unknown + 45 Gy  3 cycles + 45 Gy  4 cycles + 45 Gy  5 cycles + 45 Gy  2 cycles + 50.4 Gy  4 cycles + 50.4 Gy  Unknown + 50.4 Gy  Unknown + 40 Gy  Unknown + Unknown | 5  1  1  1  1  1  12  10  24 | 56 |
|  | Cisplatin + Taxol + RT | Unknown + 40 Gy  4 cycles + Unknown | 2  1 | 3 |
|  | Cisplatin + Paclitaxel + RT | 3 cycles + 45 Gy | 1 | 1 |
|  | Paclitaxel + Carboplatin + Pertuzumab + Trastuzumab + RT | 5 cycles + 41.4 Gy  Unknown +41.3 Gy | 1  1 | 2 |
|  | Cisplatin + Docetaxel + 5FU + RT | Unknown + 45 Gy  Unknonw+ Unknown | 2  1 | 3 |
|  | CDDP + 5FU + RT | Unknown + Unknown | 7 | 7 |
|  | Carboplatin + Taxol + RT | Unknown+ Unknown | 3 | 3 |
|  | CDDP + Docetaxel + 5FU + RT | Unknown + Unknown | 2 | 2 |
|  | Cisplatin + Folic acid + RT | 4 cycles + 50 Gy | 1 | 1 |
|  | Cisplatin + 5FU + Epirubicin + RT | Unknown + Unknown | 1 | 1 |
|  | Paclitaxel+ Carboplatin (CROSS) + RT | 5 cycles **+** 41.4 Gy | 213 | 213 |
|  | Paclitaxel+ Carboplatin + RT | 8 cycles + 45 Gy  5 cycles + 45 Gy  Unknown + 50/60 Gy | 1  1  1 | 3 |
|  | Carboplatin + 5FU + RT | 2 cycles + Unknown  Unknown + Unknown | 1  2 | 3 |
|  | Cisplatin + Docetaxel + 5FU (DCF/TCF) + RT | 4 cycles **+** 45 Gy  Unknown + 50.4 Gy | 1  1 | 2 |
|  | DDP + 5FU + RT | Unknown + 54/60 Gy  Unknown + 45 Gy  Unknown + 50 Gy  Unknown + Unknown | 1  2  1  1 | 5 |
|  | Epirubicin + Cisplatin + Capecitabin + Cetuximab + RT | Unknown + Unknown | 1 | 1 |
|  | 5FU + Leucovorin + Oxaplatin **(**FLOX) + RT | 4 cycles + 46 Gy  4 cycles + 50.4 Gy  5 cycles + 50.4 Gy  Unknown + 50.4 Gy | 1  2  1  1 | 5 |
|  | FOLFOX + RT | 3 cycles + 45Gy  4 cycles + 45 Gy  Unknown + 45 Gy | 1  1  1 | 3 |
|  | Oxaliplatin + Capecitabine + RT | Unknown + Unknown | 1 | 1 |
|  | Oxaplatin + 5 FU + RT | 3 cycles +2-46 Gy  Unknown+ 2-46 Gy | 2  1 | 3 |
|  | Taxol + 5FU + Cisplatin + RT | 3 cycles + 45 Gy  Unknown + 45 Gy  Unknown + 40 Gy  4 cycles + 40 Gy  3 cycles +50 Gy | 1  1  1  1  1 | 5 |
|  | Carboplatin + RT | Unknown + 45 Gy | 1 | 1 |
|  | Unknown | - | 25 | 24 |
| Total |  |  |  | **349** |
|  |  |  |  |  |

5-Fu=5-Fluorouracil, RT=radiotherapy, Gy=gray, TCF or DCF=docetaxel/cisplatin/5-fluorouracil, CDDP or DDP=Cisplatin, FLOX= Fluorouracil/Leucovorin/Oxaplatin, FOLFOX=folinic acid/fluoracil/oxiplatin

## ***Table S2.*** Difference in means for EORTC QLQ-C30 and EORTC QLQ-OG25 domains between reference population, patients with perioperative chemotherapy (pCT) and patients with neoadjuvant chemoradiotherapy (nCRT).

| A. EORTC QLQ-C30 | | General reference population | Patients with perioperative chemotherapy  n=216 | Patients with neoadjuvant chemoradiotherapy  n= 349 | Between general population and pCT group | Between general population and nCRT group | |
| --- | --- | --- | --- | --- | --- | --- | --- |
|  | | **Mean** | **Mean** | **Mean** | **Difference in means** | **Difference in means** | |
| Global health | | 71.2 | 71.7 | 74.0 | 0.5 | 2.8 | |
| **Functioning** |  | | | | | | |
| Physical Functioning | | 89.8 | 82.7 | 82.9 | -7.1 | -6.9 | |
| Role functioning | | 84.7 | 80.0 | 80.7 | -4.7 | -4.0 | |
| Emotional Functioning | | 76.3 | 79.3 | 83.7 | 3.0 | 7.4 | |
| Cognitive functioning | | 86.1 | 82.0 | 84.7 | -4.1 | -1.4 | |
| Social functioning | | 87.5 | 77.6 | 83.8 | -9.9 | -3.7 | |
| **Symptoms** |  | | | | | | |
| Fatigue | | 24.1 | 30.9 | 27.2 | 6.7 | 3.1 | |
| Nausea and vomiting | | 3.7 | 12.4 | 12.4 | 8.7 | 8.7 | |
| Pain | | 20.9 | 16.2 | 13.1 | -4.7 | -7.8 | |
| Dyspnoea | | 11.8 | 21.8 | 21.5 | **10.0** | 9.7 | |
| Insomnia | | 21.8 | 26.9 | 20.4 | 5.1 | -1.4 | |
| Appetite loss | | 6.7 | 17.7 | 15.5 | **11.0** | 8.8 | |
| Constipation | | 6.7 | 13.0 | 10.0 | 6.3 | 3.3 | |
| Diarrhea | | 7.0 | 20.3 | 13.7 | **13.3** | 6.7 | |
| **Financial** |  | | | | | | |
| Financial difficulties | | 9.5 | 14.1 | 12.9 | 4.6 | 3.4 | |
| B. EORTC QLQ-OG25 | | General reference population | Patients with perioperative chemotherapy  n=216 | Patients with neoadjuvant chemoradiotherapy  n= 349 | Between general population and pCT group | | Between general population and nCRT group |
|  | | **Mean** | **Mean** | **Mean** | **Difference in means** | | **Difference in means** |
| **Multi Item** | |  |  |  |  | |  |
| Dysphagia | | 0.8 | 10.5 | 9.2 | 9.7 | | 8.4 |
| Eating restrictions | | 2.9 | 23.3 | 21.0 | **20.4** | | **18.1** |
| Reflux | | 6.7 | 30.0 | 24.1 | **23.3** | | **17.4** |
| Odynophagia | | 1.5 | 12.3 | 8.5 | **10.8** | | 7.0 |
| Pain and discomfort | | 7.6 | 19.6 | 14.5 | **12.0** | | 6.9 |
| **Single Item** | |  |  |  |  | |  |
| Eating with others | | 1.3 | 12.0 | 13.5 | **10.7** | | **12.2** |
| Dry Mouth | | 11.5 | 24.0 | 20.1 | **12.5** | | 8.6 |
| Trouble with taste | | 2.6 | 14.3 | 13.1 | **11.7** | | **10.5** |
| Trouble swallowing saliva | | 1.3 | 5.6 | 6.5 | 4.3 | | 5.2 |
| Chocked when swallowing | | 3.7 | 9.9 | 10.8 | 6.2 | | 7.1 |
| Trouble with coughing | | 13.7 | 30.6 | 29.2 | **16.9** | | **15.5** |
| Trouble talking | | 2.2 | 8.3 | 10.3 | 6.1 | | 8.1 |
| Weight loss | | 1.8 | 18.4 | 18.5 | **16.6** | | **16.7** |

In bold values that are clinically relevant $difference in means\geq10$ , pCT = perioperative chemotherapy, nCRT = neoadjuvant chemoradiotherapy
